# Influence of 99m-Tc-Nanocolloid Activity Concentration on Sentinel Lymph Node Detection in Endometrial Cancer: A Quantitative SPECT/CT Study

**DOI:** 10.3390/diagnostics10090700

**Published:** 2020-09-16

**Authors:** Samine Sahbai, Francesco Fiz, Florin Taran, Sara Brucker, Diethelm Wallwiener, Juergen Kupferschlaeger, Christian La Fougère, Helmut Dittmann

**Affiliations:** 1 Department of Nuclear Medicine and Molecular Imaging, University Hospital Tuebingen, 72076 Tuebingen, Germany; sam.sahbai@gmail.com (S.S.); francesco.fiz.nm@gmail.com (F.F.); juergen.kupferschlaeger@med.uni-tuebingen.de (J.K.); Christian.LaFougere@med.uni-tuebingen.de (C.L.F.); 2 Gynecology and Obstetrics, University Hospital Tuebingen, 72076 Tuebingen, Germany; florin-andrei.taran@usz.ch (F.T.); sara.brucker@med.uni-tuebingen.de (S.B.); diethelm.wallwiener@med.uni-tuebingen.de (D.W.); 3 Cluster of Excellence iFIT (EXC 2180) “Image Guided and Functionally Instructed Tumor Therapies”, University of Tuebingen, 72076 Tübingen, Germany; 4 German Cancer Consortium (DKTK), Partner Site Tuebingen, 72076 Tuebingen, Germany

**Keywords:** sentinel lymph node, quantitative combined single photon emission tomography/computed X-ray tomography (SPECT/CT), SPECT/CT, quantitation, endometrial cancer, nanocolloid

## Abstract

This study evaluates quantitative combined single photon emission tomography/computed x-ray tomography (SPECT/CT) to assess the influence of radiotracer concentration on detection of sentinel lymph nodes (SLN) in endometrial cancer (EC). Eighty EC patients underwent pericervical 99m-Tc-nanocolloid injection followed by SPECT/CT. The subgroup of patients with failed SLN detection in SPECT/CT (*n* = 20) was compared to match-paired patients showing at least two SLNs. Results of intraoperative gamma probe measurements and quantitative SPECT/CT were used for comparison. In patients with detection failure, the percentage of injected dose, absolute activity, and volume of the injection site were significantly lower (30 ± 24% vs. 55 ± 19%; *p* = 0.01, 43 ± 36 MBq vs. 73 ± 33 MBq; *p* = 0.04, and 183 ± 164 mL vs. 266 ± 164 mL; *p* = 0.03) while mean activity concentration in liver, spleen, and bone marrow was significantly higher (*p* = 0.02). Activity concentration (>33 KBq/mL) and absolute activity (>73 MBq) of injection areas correlated with successful intraoperative SLN detection. In a subgroup of 19 SLNs, a correlation between SPECT/CT-derived uptake and intraoperative count rate was found (R^2^ = 0.8; *p* < 0.001). SLN detection in EC patients depended on high radiotracer activity at the cervical injection site. Quantitative SPECT/CT could predict successful intraoperative SLN detection and may help to optimize injection technique.

## 1. Introduction

Endometrial cancer (EC) is the third most frequent cancer in women in the USA and the fourth in Europe [1,2,3]; its incidence is continuously increasing [3]. In the early stage of gynecological cancers, the use of sentinel lymph node (SLN) mapping can potentially provide prognostic information, detect micro-metastases, and avoid systematic lymphadenectomy. Recently, SLN mapping was included into the US American guidelines for EC treatment [4]. However, it remains to be considered as an experimental approach according to the European guidelines due to the lack of large prospective studies. Therefore, larger studies are desirable to confirm the usefulness of routine SLN mapping in EC [5]. In case of intermediate/high EC recurrence risk according to the FIGO tumor classification, a bilateral pelvic and para-aortic lymphadenectomy would be preferred [6,7]. Unfortunately, complete lymphadenectomy has potential post-operative adverse effects such as lymphocele and lower-extremity lymphedema [8,9]. On the other hand, avoiding surgical lymph node staging could result in under-treatment [10]. Thus, the use of SLN mapping might obviate the need of a complete lymphadenectomy and also improve surgical staging while maximizing patients’ quality of life, similarly for breast or cervical cancer [11,12,13].

According to the literature, the detection rate of SLN mapping using Tc-99m-labelled nanocolloids ranges between 78% and 81% [14,15], which is inferior to the overall detection rates (up to 98.6%) that have been achieved in cervical cancer using combined single photon emission tomography/computed x-ray tomography (SPECT/CT) imaging [16]. Our previous experience showed a SPECT/CT derived SLN detection rate of 83% in EC when the pericervical tracer injection technique was used [17]. High venous drainage (as indicated by intense bone marrow tracer accumulation) was identified as the most important factor of SLN detection failure [18]. On the other hand, liver/spleen uptake and other parameters like tumor stage and patients’ physiological characteristics were not associated with detection failure [18]. Similar to this, neither patient’s body mass index nor tumor histology and/or tumor grade were predictive of detection failure in a meta-analysis [14].

A poor lymph node detection rate is likely to prevent acceptance of the SLN procedure in clinical routine. Further improvements are thus necessary to identify and possibly eliminate sources of error, so to make SLN mapping as reliable and as reproducible as possible. However, even though SPECT/CT is a growing technique used for an increasing number of clinical indications, it suffers from limitations when it comes to comparing uptake intensity between patients [19]. Quantitative approaches to SPECT and SPECT/CT have been attempted in different clinical scenarios, showing the capability to provide better results when compared to plain qualitative analysis [19,20,21]. Recently, attenuation correction combined with scatter correction and resolution recovery allowed for an absolute quantification of SPECT data comparable to positron emission tomography (PET) imaging [22]. This approach was shown to be accurate in a number of phantom studies [23,24]. Moreover, intra- and inter-observer agreement was shown to be highly reproducible by the use of commercially available semi-automatic segmentation software [25].

The current study was conceived to investigate whether absolute quantitation of 99m-Tc-nanocolloid uptake may gain new insights into SLN detectability in EC. Our SPECT/CT analyses included activity concentration estimates in lymph nodes at the tracer injection site and also in non-target areas such as organs and the entire field-of-view (FOV). The aim of the present study was to test whether this quantitative approach might improve our understanding of nanocolloid pathophysiology by identifying thresholds of activity concentration that could predict SLN detection.

## 2. Materials and Methods 

### 2.1. Patient Cohort

All patients with endometrial cancer who underwent surgery and SLN biopsy in 2015 and 2016 at our institution were consecutively included. Scintigraphic SLN mapping was performed one day before surgery after injection of a total of 0.8 cm^3^ 99m-Tc-nanocolloid into four cervical sub-epithelial injection points supplemented by an injection of 0.5 cm^3^ saline. This technique was selected on the basis of previous results, as it was associated with a favorable SLN detection rate [17]. This study was approved by the institutional review board of our university hospital. All patients provided written informed consent.

In the current study group, 20/80 patients had no evidence of SLN nodes at visual image analysis. These patients were compared to a subgroup of 20 match-paired patients selected with computed randomization among individuals with at least two detected SLNs in SPECT/CT.

### 2.2. SPECT/CT Imaging and Quantification

The imaging procedure followed 2–6 h after injection of 99m-Tc-nanocolloid (mean 261±19 MBq) using a hybrid SPECT/CT camera, which allows quantitative measurements (Discovery 670 Pro^®^, GE Healthcare, Chicago, IL, USA). The FOV included the area from the pelvis to the caudal liver. Acquisition parameters were as follows: 30 steps at 6 °C intervals and 15 s acquisition time per step, matrix 128 × 128, and voxel size 4.42 mm^3^. A low dose CT scan (automated dose adaptation 10–80 mA, 120 kV, slice thickness 2.5 mm) was performed for attenuation correction and anatomical mapping. The SPECT images were reconstructed using ordered subset expectation maximization (OSEM; two iterations, 10 subsets). 

Imaging findings were analyzed by two experienced nuclear medicine physicians. SLN detection was defined by the visualization of hot-spots in the lymph node (LN) areas using SPECT/CT. Absolute quantitative SPECT/CT data were generated by use of commercial software (Q.Metrix^®^, GE Healthcare, Chicago, IL, USA) including corrections for motion, attenuation, scatter, and decay, as well as resolution recovery. Quantitative measurements were based on the following input parameters: gender, weight, and height of patient; measured and injected activity; and well as system sensitivity. 

Semi-automatic segmentation based on a manually defined threshold (80% of maximum) from a seed point was used for volume-of-interest (VOI). These standardized VOIs comprised the following organs or areas: each detected SLNs, liver, spleen, injection site at the cervix uteri, and the total FOV. Additional standardized spherical VOIs of 20 mL were placed in each lumbar vertebra (from L2 to L5) to measure bone marrow uptake. Quantities for percentage of injected 99m-Tc-nanocolloid dose (%ID), mean of activity concentration (in kBq/mL), and absolute activity (in MBq) in assessed VOIs were calculated using the Q.Metrix^®^ software. Liver and spleen were not completely included in the SPECT/CT FOV in most cases, thus only aliquot proportions, not representative of total organ volumes, were accessible for quantification. For this reason, only radioactivity concentration, but not total activity, was available in these organs. Finally, presence of bone marrow uptake, liver and spleen uptake, as well as presence of radioactivity in the abdominal area and outside of the patient in the crotch area were visually analyzed in SPECT/CT.

### 2.3. Surgery

Surgical staging was performed the day after lymphoscintigraphy. Each SLN was localized and identified using a handheld gamma probe (Neoprobe^®^, Models 1017 and 1100, Dublin, OH, USA). SLN detection, histopathological results, and tumor staging were collected.

### 2.4. Statistics

Patient characteristics, histological results and surgery reports were collected and analyzed. Mann–Whitney U-test, receiver operating characteristic (ROC) analysis and multivariate analysis of variance (MANOVA) were performed with SPSS Statistics^®^ software application, version 21 (IBM Inc., Armonk, NY, USA). A *p*-value <0.05 was considered as statistically significant.

## 3. Results

### 3.1. General Characteristics of the Populations

In 20/80 patients (25%) no SLN could be visualized on SPECT/CT images. Seventeen of the 80 patients (21%) had only one detected SLN. A total of 75 SLNs were identified in the control subgroup of randomly selected patients (*n* = 20) with at least two detectable SLNs (*n* = 43). Five of the randomly selected 20 patients had unilateral SLN detection only. Clinical and patient characteristics were comparable in both groups and are resumed in Table 1.

### 3.2. Quantification in SPECT/CT and Multivariate Analysis

**Figure 1:**
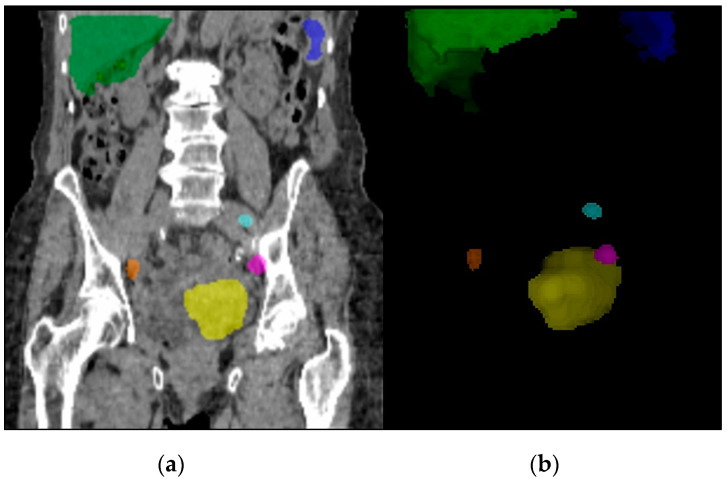
Segmented volume-of-interests (VOIs) for SPECT/CT quantification in an endometrial cancer (EC) patient. The case study shows VOIs for liver (green), spleen (blue), injection site (yellow), and three bilateral iliac SLNs (other colors) in coronal low dose CT (**a**) and in tridimensional representation of the regions (**b**).

**Figure 2:**
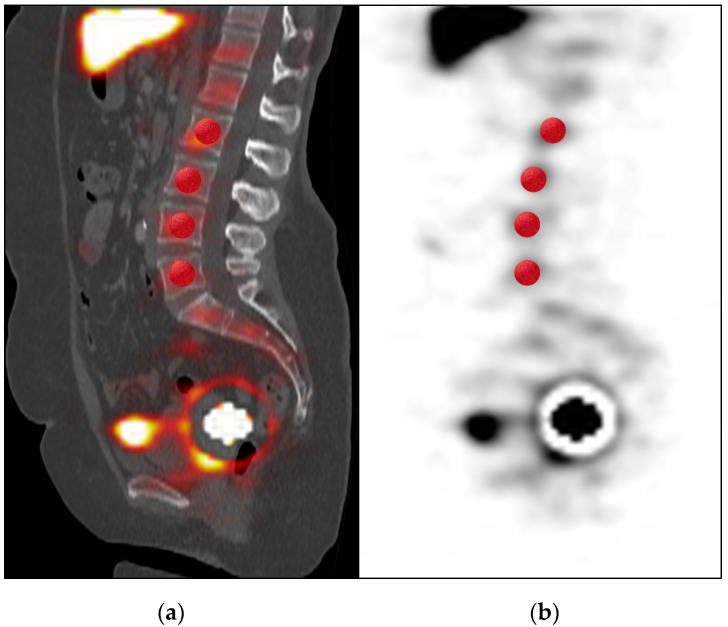
Spherical VOIs (red) placed in lumbar vertebrae (L2 to L5) shown in sagittal SPECT/CT (**a**) and SPECT (**b**) for bone marrow uptake measurement.

Examples of VOI allocations in SPECT/CT are presented in Figure 1 and Figure 2. From the multivariate analysis, significant predicting factors of failed SLN detection in SPECT/CT were liver and spleen activity concentration, which was higher in the group without detected SLN compared to the control cohort (mean 26 ± 20 vs. 11 ± 8 kBq/mL and 11 ± 9 vs. 5 ± 4 kBq/mL, respectively; *p* = 0.02; see Table 2; VOIs mean volume 937 ± 126 mL for liver and 470 ± 90 mL for spleen). Moreover, mean radiotracer uptake in bone marrow from lumbar vertebrae was also significantly higher in the case of detection failure (mean 1.8 ± 1.2 vs. 1.0 ± 1.0 kBq/mL; *p* = 0.02). Regarding the injection site, a significantly higher percentage of injected 99m-Tc-nanocolloid dose (%ID), absolute activity, and volume of the injection depot was measured in the group with successful SLN detection in SPECT/CT (55 ± 19% vs. 30 ± 24%; *p* = 0.01, 73 ± 33 vs. 43 ± 36 MBq; *p* = 0.04, and 266 ± 164 vs. 183±164 mL; *p* = 0.03, respectively). Moreover, we observed a significantly lower overall %ID in the SPECT/CT-FOV in the case of detection failure (62 ± 26% vs. 77 ± 13%; *p* = 0.04). No significant relationship with SLN detection was found for other quantitative variables, in particular for initial injected activity or time elapsed after injection.

### 3.3. Quantitative SPECT/CT Analysis of Detected SLNs

**Figure 3:**
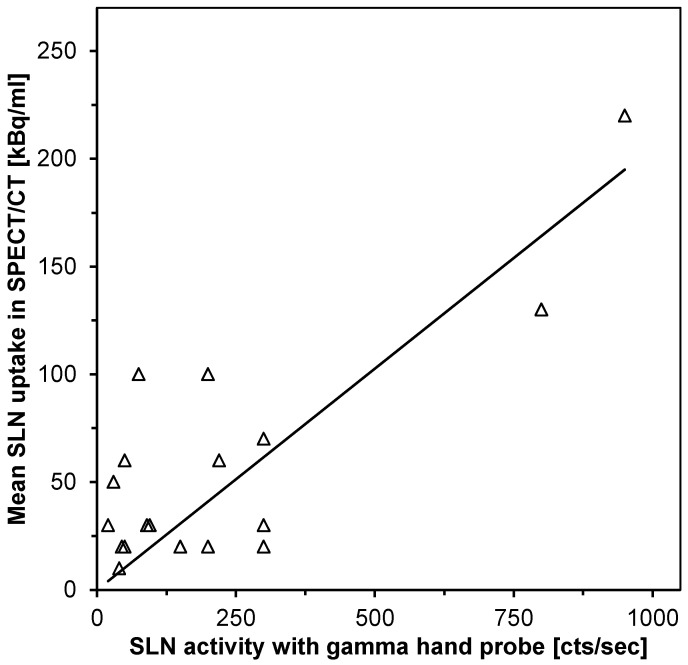
Linear representation of SLN activity as measured by quantitative SPECT/CT and by gamma hand probe during surgery (*n* = 19; R^2^ = 0.8; *p* < 0.001).

Of the 75 detected SLNs due to prominent uptake in SPECT, only 20 could be delineated on the corresponding low-dose CT scans (diameter from 4 to 20 mm, mean 7.6 ± 3.8mm). Partial volume correction was not performed. A correlation was identified between SPECT/CT activity concentration and the reported counts per sec activity of 19 SLNs measured during surgery by means of hand-held gamma probe (R^2^ = 0.8; *p* < 0.001; Figure 3).

### 3.4. Visual Analysis of SPECT/CT

Presence of bone marrow uptake was observable in approximately one third of patients with SLN detection failure but was never witnessed in patients with successful SLN detection (35% vs. 0%; *p* = 0.01; Table 2). Similarly, a higher prevalence of aberrant radioactivity in the crotch area was detected in the cohort with detection failure (45% vs. 15%; *p* = 0.04; Table 2). Of note, bone marrow uptake was always associated with liver/spleen uptake.

### 3.5. Analysis of Parameters Influencing SLN Detection During Surgery

Intra-operative SLN detection with gamma hand probe was successful in 22 out of the selected 40 patients (17 from the SPECT/CT-detection cohort and 5 from the non-detection cohort in SPECT/CT; see Table 2). These 22 patients with at least one detected SLN during surgery had significantly higher activity concentration and absolute measured activity in SPECT/CT at the injection site (35 ± 16 vs. 22 ± 7 kBq/mL; *p* = 0.001 and 73 ± 33 vs. 43 ± 36 MBq; *p* < 0.05 respectively). In one of the 18 remaining patients with failed intra-operative SLN detection, there was a gamma hand probe dysfunction. This patient was excluded from statistical analysis (see Table 3). Using ROC-analysis, the estimated cut-off value for SLN detection during surgery was 33 kBq/mL and 73 MBq for activity concentration and absolute activity in the application site, respectively. The quantified activity within liver, spleen and bone marrow activity as well as other parameters were not associated with SLN detection at surgery.

## 4. Discussion

To the best of our knowledge, this is the first study on quantitative SPECT/CT dedicated to SLN procedures. The use of this technique allowed for identifying predictive variables for SLN detection both at SPECT/CT imaging and at surgery. Quantitative analysis enabled us to establish a threshold activity at the injection site that can predict successful intra-operative SLN identification. This confirms the results of a previous analysis, which stated that successful sentinel node detection is dependent on an adequate tracer depot at the injection site [23]. Moreover, the current analysis demonstrated a correlation between the magnitude of activity in lymph nodes in SPECT/CT and the intensity of the gamma hand probe signal from the extracted nodes. Thus, quantitative SPECT/CT may be used to inform the surgical team about the precise location of the node as well as the probable signal intensity that is to be expected during surgery. In particular, it might allow adapting the surgical procedure in case of SLNs with a weak signal or in hard-to-reach positions and could predict the likelihood of extended lymphadenectomy in case of SLN detection failure during surgery as an alternative. Even a re-injection of radiotracer might be considered when low activity at the application site is measured in quantitative SPECT/CT.

The use of quantitative SPECT/CT in the current study confirmed that high bone marrow uptake was associated with SLN detection failure [18]. Moreover, quantification showed that detection failure was also correlated with increased uptake in liver and spleen. As discussed before, this phenomenon can be explained by considerable venous drainage of 99m-Tc-nanocolloid, which in turn reduces the quantity of radiotracer available for the lymphatic one.

Higher radiotracer uptake retained at the application site, measured as absolute activity (in MBq) and percentage of injected activity (%ID), was associated with better SLN detection in SPECT/CT. Similar to this, we also found a significant correlation between activity concentration and absolute activity at the injection site with intra-operative recognition of SLNs. Taken together, our findings suggest that a significant radioactivity reservoir at the injection site is crucial for SLN detectability. Both loss of radiotracer outside of the patient and venous drainage could reduce available radioactivity from this pool and possibly impair the pressure-based mechanisms, which are needed for lymphatic drainage in valveless fifth- and fourth-order lymphatic vessels [26]. Until now, there is no standardized injection protocol established for SLN mapping in EC, and two to four pericervical injections have been reported in the literature [4]. Currently, with four sub-epithelial injections used throughout, a high distribution volume of the radiotracer at the application area was related to successful SLN detection. Given that the tracer distribution volume was linked to the number of injection depots, our results may imply to favor four over two injections. Above that, the depth of injections might influence retention of the radiopharmaceutical. These results should encourage studies with varied numbers of injections and application depths. Above these functional aspects, detection failure can be progressively avoided as the operator expertise increases [27,28].

Some limitations are to be mentioned in our small cohort study. A VOI of the full skeleton for bone marrow measurements was not possible due to the limited FOV in SPECT/CT. Therefore, the quantitative analysis was performed with a standardized representative segmentation in the lumbar vertebrae (four VOIs in L2 to L5). According to an earlier phantom study using the same gamma camera system and software algorithm, absolute radioactivity quantification was feasible for large sphere volumes but not reproducible for sphere volumes smaller than 5 mL, where true activity was at least 20% higher than measured activity [29]. This finding is attributed to partial volume effects, which underestimate quantitative measurements for small lesions. In principle, partial volume correction is especially critical in lymph nodes and should be applied for reliable quantification of all SLNs smaller than 20 mm. However, in our study, only a very limited number of SLNs could be delineated on non-contrast-enhanced low dose CT so that efficient recovery correction was not possible. As a result, estimated 99m-Tc-nanocolloid uptake values of SLNs can only be considered semi-quantitative in this study. This limitation should not affect quantitation of organs and application sites where larger VOIs were used. In fact, for large solid organs like the liver, there was only a slight overestimation from the true radioactivity in a recent phantom study [24]. 

Currently, disagreement between lymphoscintigraphy and intra-operative SLN detection was seen in some patients. In cases with SLNs detected only during surgery, prolonged tracer drainage may have occurred as discussed previously [18]. On the other hand, if SLNs were detected solely in SPECT/CT, lymph node-to-background contrast might have been too low for identification at surgery [30]. 

The present study shows that additional quantitation of SPECT/CT data is feasible in clinical routine and may help to better understand the biodistribution of the injected radiotracer. In particular, quantitation could be used in clinical studies on different injection techniques and for the evaluation of new radiotracers aimed to increase the sensitivity of SLN procedures. Earlier work has demonstrated, that SLNs with massive metastatic involvement may display little or no radioactivity uptake at all, possibly increasing the false negative rate of this method [12]. A combined pre-operative algorithm using whole-body tumor imaging (e.g., 18-F-Fluorodeoxyglucose (FDG)-PET/CT) and 99m-Tc-nanocolloid SPECT/CT for SLN mapping to detect non visible lymph node micro-metastases, may improve the accuracy of lymph node metastasis detection and may ultimately avoid unnecessary systematic bilateral lymphadenectomy frequently associated with morbidity [31,32].

## 5. Conclusions

Quantitative SPECT/CT demonstrated that both high activity and high distribution volume at the cervical application site are predictive of successful SLN identification in endometrial cancer. Our study suggests that injections must ensure retention of a substantial amount of tracer at the application area. Quantitative SPECT/CT might be helpful for optimizing injection techniques and for evaluating different SLN targeting radiopharmaceuticals.

## Figures and Tables

**Table 1:** Patient baseline characteristics.

SPECT/CT Detection	No SLN Detected	SLN Detected
Number of patients	20	20
Mean age (range)	63 (36–88)	63 (35–79)
Mean BMI (range)	29.3 (17–39)	29.6 (21–42)
Mean activity (MBq)	263	260
Mean time p.i. h (range)	3:46 (2:20–5:41)	3:36 (2:20–5:51)
**Histological Type**		
Endometrioid	19	18
Serous	0	2
Carcinosarcoma	1	0
**Grade**		
G1	9	12
G2	9	5
G3	2	3
**FIGO**		
IA	14	14
IB	3	4
II	2	1
III	1	1
IV	0	0

SPECT/CT = combined single photon emission tomography/computed x-ray tomography; SLN = sentinel lymph node; BMI = body mass index; FIGO = Fédération Internationale de Gynécologie et d’Obstétrique; p.i. h = hours post injection.

**Table 2:** Parameters significantly affecting pre-operative SLN detection by SPECT/CT.

SPECT/CT Detection	No SLN Detected	SLN Detected	*p*-Value
Patients, *n*	20	20	
Quantitative SPECT/CT parameters			
Liver mean uptake (kBq/mL)	26	11	0.02
Spleen mean uptake (kBq/mL)	11	5	0.02
Bone marrow mean uptake (kBq/mL)	1.8	1.0	0.02
Absolute activity at injection site (MBq)	43	73	0.04
VOI of the injection site (ml)	183	266	0.03
%ID of the injection site %ID of the whole FOV	30% 62%	55% 77%	0.01 0.04
**Visual SPECT/CT parameters**			
Presence of bone marrow uptake (%)	35%	0%	0.004
Radioactivity in crotch area (%)	45%	15%	0.04

**Table 3:** SPECT/CT derived quantitative parameters significantly affecting intra-operative SLN detection using gamma hand probe.

Gamma Hand Probe Detection	No SLN Detected	SLN Detected	*p*-Value
Patients, *n*	17 ^#^	22	
Quantitative SPECT/CT parameters			
Injection site mean uptake (kBq/mL)	22	35	0.001
Absolute activity at injection site (MBq)	43	73	0.02

^#^ 1 patient excluded from 18 patients due to gamma probe failure.
